# Care of Infants

**Published:** 1882-06

**Authors:** 


MONTHLY.

E. H. GIBBS, A. M., M. D., EDITOR.

FOUNDED IN 1854.

### CARE OF INFANTS.

The first two years of life is the period of infancy; and it is also the period of greatest mortality.

The things most essential to the infant are pure air, appropriate and seasonable clothing and proper food.

Hence, the room occupied by the infant should be well ventilated and kept at an equable and comfortable temperature. Its bed should be an ordinary hair mattress, with cotton sheets and woolen blankets. Its clothing should be carefully adapted to the changing seasons, to protect it from extremes of heat and cold.

It should wear a soft flannel wrapper next the skin, thin in summer, heavier in winter. If, during infancy, a flannel band is also worn around the middle of the body to protect the bowels, and woolen stockings long enough to cover the knees, they will secure almost entire exemption from attacks of colic and diarrhea.

Diet of Infants.—The mother's milk is the most appropriate food for infants; provided the mother is in perfect health and not liable to develop, through inheritance, any disease of body or mind. I have given the subject careful attention during many years, and do not believe that in the United States there is more than one mother in ten who should nurse her children. Under the most favorable circumstances, the mother, after a two years' *siege* will look and feel five years older, and the child will seldom be as strong, healthy and free from attacks of illness as when "raised on the bottle" with milk from cows or goats.

The reason is, that our modern life is so artificial, so full of nervous excitement and wear and tear and whirl, that nature's designs are frustrated in this respect, and that which was intended to nourish the infant becomes a source of weakness and disease.

